# Healthberry 865^®^ and Its Related, Specific, Single Anthocyanins Exert a Direct Vascular Action, Modulating Both Endothelial Function and Oxidative Stress

**DOI:** 10.3390/antiox10081191

**Published:** 2021-07-26

**Authors:** Albino Carrizzo, Rosario Lizio, Paola Di Pietro, Michele Ciccarelli, Antonio Damato, Eleonora Venturini, Patrizia Iannece, Eduardo Sommella, Pietro Campiglia, Philipp Ockermann, Carmine Vecchione

**Affiliations:** 1 Cardiovascular Research Unit, Department of Medicine and Surgery, University of Salerno, 84081 Baronissi, Italy; albino.carrizzo@gmail.com (A.C.); pdipietro@unisa.it (P.D.P.); mciccarelli@unisa.it (M.C.); piannece@unisa.it (P.I.); 2 Laboratory of Vascular Physiopathology—I.R.C.C.S. Neuromed, 86077 Pozzilli, Italy; antonio.damato85@gmail.com (A.D.); eleonora.venturini94@libero.it (E.V.); 3 Evonik Operations GmbH, Rodenbacher Chaussee 4, 63457 Hanau, Germany; rosario.lizio@evonik.com; 4 Department of Pharmacy, University of Salerno, 84084 Fisciano, Italy; esommella@unisa.it (E.S.); pcampiglia@unisa.it (P.C.); 5 Institute for Tissue Engineering and Regenerative Medicine, Universität Würzburg, Josef-Schneider Straße 2, 97080 Würzburg, Germany; philipp.ockermann@uni-wuerzburg.de

**Keywords:** anthocyanins, cardiovascular, endothelium, oxidative stress

## Abstract

In recent years, epidemiological studies have identified a relationship between diet and cerebro–cardiovascular disease (CVD). In this regard, there is a promising dietary group for cardiovascular protection are polyphenols, especially anthocyanins. Vascular reactivity studies were performed using Healthberry 865^®^ and constituent single anthocyanins to characterize vasomotor responses; immunofluorescence analysis with dichlorofluorescein diacetate and dihydroethidium were used to evaluate nitric oxide and oxidative stress; lucigenin assay was used to measure NADPH oxidase activity; and gel electrophoresis and immunoblotting were used to dissect the molecular mechanisms involved. We demonstrated that Healthberry 865^®^ exerts an important vasorelaxant effect of resistance artery functions in mice. Its action is mediated by nitric oxide release through the intracellular signaling PI3K/Akt. Moreover, behind its capability of modulating vascular tone, it also exerts an important antioxidant effect though the modulation of the NADPH oxidase enzyme. Interestingly, its cardiovascular properties are mediated by the selective action of different anthocyanins. Finally, the exposure of human dysfunctional vessels to Healthberry 865^®^ significantly reduces oxidative stress and improves NO bioavailability. Although further investigations are needed, our data demonstrate the direct role of Healthberry 865^®^ on the modulation of vasculature, both on the vasorelaxation and on oxidative stress; thus, supporting the concept that a pure mixture of anthocyanins could be helpful in preventing the onset of vascular dysfunction associated with the development of CVD.

## 1. Introduction

It is estimated that about 18 million people worldwide died from cardiovascular disease (CVD) in 2015, and more people die every year from CVD than from any other cause. Due to the impossibility of acting on non-modifiable cardiovascular risk factors, such as age, gender, genetics, and ethnicity, pharmacological therapy remains a unique validated clinical approach that is able to fight CVD incidence and progression (although leading to a dramatic increase in global spending) [1,2]. Thus, discovering new substances that are able to evoke cardiovascular protection is imperative. Protective or preventive substances are of high interest, such as arterial stiffness, a predictor for CVD—it is not only reversible via diet and exercise, but also dietary components, such as resveratrol, as evidenced by both in-vivo [3] and ex-vivo approaches [4].

In recent years, epidemiological studies have identified a relationship between diet and CVD; though there is still considerable scientific uncertainty about the relationship between specific dietary components and cardiovascular risk [5,6]. One promising dietary group for cardiovascular protection are polyphenols, especially flavonoids, as they are inversely associated with blood pressure and a lower risk of hypertension [7]. In this regard, anthocyanins, natural pigments belonging to the flavonoid family, are widely distributed in the human diet, such as in beans, fruits, vegetables, and red wine [8]. It is well-accepted that these natural compounds present in fruits and plant-derived-foods are relevant because of their potential health-promoting effects, as suggested by the available experimental and epidemiological evidence [9,10,11]. For this reason, interest in the biochemistry and biological effects of anthocyanins has increased substantially during the last decade. Several experimental studies have reported that anthocyanins exert a positive impact on health status, as showed by their anti-inflammatory and anti-oxidative effects in a mouse model involving inflammatory bowel disease [12,13], by the regulation of glucose metabolism and insulin, or exerting anti-obesity effects in a mouse model [14,15], inhibiting, in-vitro, the growth of human breast cancer cells [16]. Concerning CVD, anthocyanins from blueberries or red wine showed improvement in flow-mediated dilation (FMD), and augmentation index in humans, as well as NO-dependent vessel relaxation in mice [17,18]. Despite their beneficial properties, the possible direct action of anthocyanins on the vasculature, both at functional and molecular levels, remains completely unknown.

Most popular products containing anthocyanins include other different biologically active compounds, such as polyphenols, making it hard to assess the selective effects of anthocyanins. Thus, in the present study, we used Healthberry 865^®^ powder (HB), which is a standardized product containing stable, high, and defined amounts of anthocyanins sourced specifically from bilberries (Vaccinium myrtillus) and blackcurrant (Ribes nigrum).

In this study, we investigated the effects of Healthberry 865^®^ and of its single anthocyanins on vascular function and oxidative stress. Moreover, we evaluated the cumulative effects by mixing the most powerful anthocyanins.

Here, for the first time, we demonstrated that HB exerts a substantial vasorelaxant effect on resistance arteries. Its action is mediated by enhancing nitric oxide release through the intracellular signaling PI3K→Akt. Moreover, it exerts an important antioxidant effect through the modulation of the NADPH oxidase enzyme. Finally, the exposure of dysfunctional human vessels to HB significantly reduces oxidative stress and improves NO bioavailability.

## 2. Materials and Methods

### 2.1. Reagents

HB and single anthocyanins, delphinidin-3-rutinoside (D3-rut), cyanidin-3-rutinoside (C3-rut), delphinidin-3-glucoside (DP3-glu), cyanidin-3-glucoside (C3-glu), petunidin-3-glucoside (PT3-glu), delphinidin-3-galactoside (DP3-gal), peonidin-3-galactoside (PEO3-gal), delphinidin-3-arabinoside (DP3-ara), malvidin-3-galactoside (MAL3-gal), malvidin-3-glucoside (MAL3-glu), cyanidin-3-galactoside (C3-gal), cyanidin-3-arabinopyranoside (C3-arapy) were obtained from Evonik Operations GmbH, Germany. Primary antibodies and horseradish peroxidase (HRP)-labeled anti-rabbit or anti-mouse fragment immunoglobulin, and enhanced chemiluminescence for Western blotting detection reagent were purchased from Amersham Biosciences. All inhibitors, powders, and solvents necessary for the preparation of the buffers were purchased from Sigma-Aldrich.

### 2.2. Vascular Reactivity Studies

Aorta, carotid, femoral arteries, and second-order branches of the mesenteric arterial tree were removed from wild type C57BL/6 mice to perform vascular studies. Vessels were placed in a wire or pressure myograph system filled with Krebs solution maintained at pH 7.4 at 37 °C in oxygenated (95% O2/5% CO2). First, an analysis of vascular reactivity curves was performed. In particular, vasoconstriction was assessed with 80 mmol/L of KCl or with increasing doses of phenylephrine (from 10^–9^ M to 10^–6^ M) in control conditions. Endothelium-dependent and -independent relaxations were assessed by measuring the dilatory responses of mesenteric arteries to cumulative concentrations of acetylcholine (from 10^–9^ M to 10^–6^ M) or nitroglycerine (from 10^–9^ M to 10^–6^ M), respectively, in vessels precontracted with phenylephrine at the dose necessary to obtain a similar level of pre-contraction in each ring (80% of initial KCl-evoked contraction). Caution was taken to avoid endothelial damage; functional integrity was reflected by the response to acetylcholine (from 10^–9^ M to 10^–6^ M).

Vascular responses were then tested, administering increasing doses of HB or single anthocyanins. Some experiments were performed in presence of selective inhibitors, such as phosphatidylinositol-4,5-bisphosphate 3-kinase inhibitor (LY274002, 10 µM,1 h—Sigma # L9908), Akt inhibitor X (Akt inh, 1 µM, 1 h—Sigma #124020), or the NOS inhibitor N-ω-nitro-l-arginine methyl ester (L-NAME, 300 µM, 30 min—Sigma #N5751), before data for dose–response curves were obtained.

### 2.3. Evaluation of NO Production by DAF

Production of NO was assessed as previously described [19]. HB (100 µg/mL) or acetylcholine (10−6 M) was administered to the mesenteric artery in the last 30 min of 4-amino-5-methylamino-2,7,-diflfluoroflfluorescein diacetate (DAF-FM) incubation, alone and after 20 min exposure to L-NAME (300 μmol/L, 30 min). Mesenteric segments were cut in 5-µm thick sections, observed under a fluorescence microscope (Zeiss Axio Observer A1—Obj. 20X), subsequently counterstained with haematoxylin and eosin, and observed under a light microscope.

### 2.4. Analysis of Total ROS Production

Dihydroethidium (DHE, Life Technologies) was used to evaluate production of reactive oxygen species (ROS) in mouse mesenteric arteries, as previously described [20]. Briefly, vessels were incubated with 5 µM of DHE for 20 min and subsequently observed under a fluorescence microscope (Zeiss Axio Observer A1—Obj. 20X). Images were acquired by a digital camera system (Olympus Soft Imaging Solutions). A second, estimation of total ROS production in mouse vessels was performed with the membrane-permeable fluorescent probe an analog of 2,7-dichlorodihydrofluorescein (DCDHF), dihydrorhodamine 123 (DHR123) (Invitrogen). After treatment, vessels were incubated with Krebs solution containing 5 μM DHR123 for 30 min at 37 °C, and then washed two times with PBS prior to fluorescence measurement using a fluorescence microplate reader (TECAN infinite 200 Pro).

### 2.5. Evaluation of NADPH-mediated O_2_^−^ Production

To determine NADPH oxidase-mediated superoxide radical (O_2_^−^) production, we used the lucigenin-enhanced chemiluminescence assay, as previously described [21]. Vessels were homogenized in a buffer containing protease inhibitors (mmol/L: 20 monobasic potassium phosphate, 1 EGTA, 0.01 aprotinin, 0.01 leupeptin, 0.01 pepstatin, 0.5 phenylmethylsulfonyl fluoride, pH 7.0). Protein content was measured in an aliquot of the homogenate by the Bradford method. The reaction was started by the addition of NADPH (0.1 mmol/L) and lucigenin (5 μmol/L) to each well. The chemiluminescence was measured using Tecan Infinite Pro M200 multimode microplate at 37 °C.

### 2.6. Gel Electrophoresis and Immunoblotting

After isolation, arteries were solubilized in lysis buffer containing 20 mmol/L Tris-HCl, 150 mmol/L NaCl, 20 mmol/L NaF, 2 mmol/L sodium orthovanadate, 1% Nonidet, 100 μg/mL leupeptin, 100 μg/mL aprotinin and 1 mmol/L phenylmethylsulfonyl fluoride. Samples were left on ice for 30 min, centrifuged at 13,000× *g* for 15 min and supernatants were used to perform Western immunoblot analysis. Total protein levels were determined using the Bradford method. Thirty μg of proteins were resolved on 8% SDS-PAGE, transferred to a nitrocellulose membrane, and immunoblotted with anti-phospho-eNOS Serine 1177 (Cell Signaling, rabbit polyclonal antibody 1:800) and with anti-total-eNOS (Cell Signaling, mouse mAb 1:1000). HRP-conjugated secondary antibodies were used at 1:3000 dilution (Bio-Rad Laboratories). Protein bands were detected by ECL Prime (Amersham Biosciences) and quantitated with ImageJ software.

### 2.7. Human Vessels Preparation and Ex-Vivo Experiments

The study protocol was approved by all local ethics committees of IRCCS Neuromed and conducted in accordance with the Declaration of Helsinki. All participants gave written informed consent. Institutional review board approval was obtained from IRCCS Neuromed (no. 20160106-1006). Vascular reactivity studies were performed on segments of human superior thyroid artery (STA) removed during bypass surgery, which represented waste material superfluous for revascularization. The vessels were supplied before the dilation procedure and vasodilator application. Care was taken during harvesting of the vessels so as not to stretch or touch the endothelial surface. The vessel species were placed immediately into cold (4 °C) Krebs Ringer solution of the following composition (mmol/L): NaCl 118, KCl 4.7, KH2PO_4_ 1.2, NaHCO_3_ 25, MgSO_4_d7H_2_O 1.2, CaCl_2_ 2.5, glucose 11.1, and disodium EDTA 0.026. The vessels were cleaned of adherent connective tissues and cut into rings 3 to 4 mm in length. Rings were suspended between 2 stainless steel L-shaped hooks in a 10-mL jacketed organ bath containing Krebs Ringer solution at 37 °C and aerated with 95% O_2_ and 5% CO_2_. One hook was fixed to a micrometric manipulator allowing adjustments in resting tension of the rings, and the other was connected to a force displacement transducer (WPI Instruments, Sarasota, FL) for the measurement of isometric force. At the end of an equilibration period of 2 h, the viability of the vessel segments was checked through the evaluation of the cumulative dose–response curves to norepinephrine (10^−9^ to 10^−6^ mol/L) and contraction to 40 mmol/L KCl, which had to be reproducible at least 2 times. To study the endothelium-dependent vasorelaxation, human vessels were precontracted with 10^−9^ to 10^−6^ mol/L of phenylephrine to obtain a contraction that corresponds to 70% to 90% of maximal contractions observed with KCl. When the constriction reached a stable plateau, increasing doses of acetylcholine (10^−9^ to 10^−6^ mol/L) were added. Cumulative dose–response curves to acetylcholine in STA were evaluated before and after incubation with HB865^®^ for 1 h at the dosage of 50 μM.

### 2.8. Statistical Analysis

Data are presented as mean ± SEM. Statistical analysis was performed by 2-way ANOVA followed by Bonferroni post hoc test. Repeated measurements were analyzed by one-way ANOVA followed by the Bonferroni post-hoc test. Differences were considered to be statistically significant at *p* < 0.05.

## 3. Results

### 3.1. Healthberry 865^®^ Evokes a Direct Vasorelaxant Action of Conduit and Resistance Arteries

**Figure 1:**
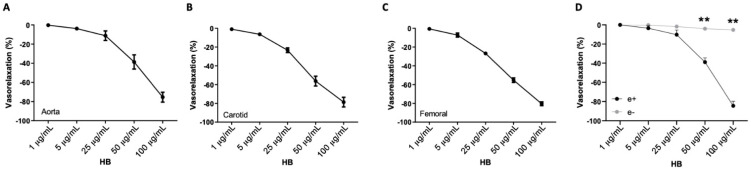
(**A**–**D**) Vascular response of phenylephrine-precontracted mice vessels to increasing doses of HB (1–100 µg/mL) on (**A**) aorta (*N* = 5), (**B**) carotid (*N* = 5), (**C**) femoral (*N* = 5), or (**D**) vascular response of phenylephrine-precontracted mice mesenteric arteries to increasing doses of HB in vessels with endothelium (e+) and without endothelium (e−) (*N* = 5). ** *p* < 0.01.

In order to evaluate the vascular properties of HB (composition and concentration are reported in Supplementary Materials; Supplementary Table S1, Supplementary Figures S1–S3), we performed a first series of experiments on different vascular districts, aorta and carotid arteries, and femoral and mesenteric arteries, which represent, respectively, the prototypes of conduit and resistance arteries. Interestingly, the administration of increasing doses of HB (1 µg/mL to 100 µg/mL) was able to exert, per se, a direct vasorelaxant action on both kinds of vascular districts. As shown in Figure 1A–D, HB evokes a dose-dependent vascular effect, and at the maximal dose used of 100 µg/mL, was able to reach ~80–90% of vasorelaxation in all studied vessels.

Based on the well-validated concept that alteration of resistance arteries exerts an important role in the development, and may contribute to the complications, of cardiovascular disease, we have focused our attention to the characterization of vascular function of HB and their relative anthocyanins on resistance artery functions in mice, involved in blood pressure regulation, the mesenteric artery [22,23,24,25].

### 3.2. The Vascular Effect of Healthberry 865^®^ Is Endothelial Nitric Oxide Synthase-Mediated

**Figure 2:**
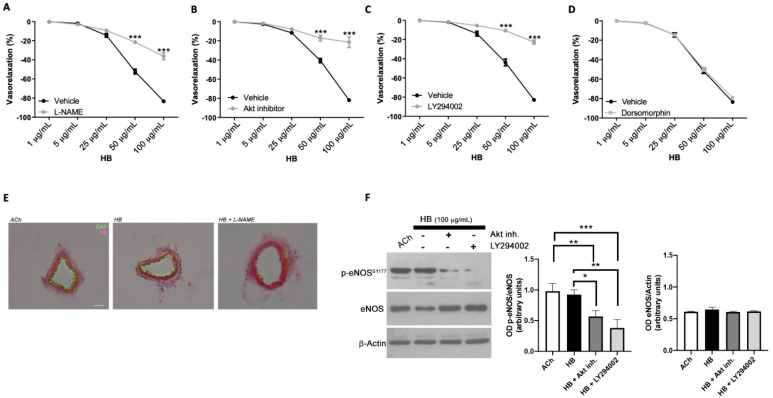
(**A**–**D**) Vascular response of phenylephrine-precontracted mice mesenteric arteries to increasing doses of HB in the presence of (**A**) L-NAME (*N* = 7), (**B**) Akt inhibitor (*N* = 7), (**C**) in the presence of PI3K inhibitor (LY294002) (*N* = 7), or (**D**) in the presence of AMPK inhibitor (dorsomorphin) (*N* = 5). (**E**) Representative high-power micrographs of 10 µm sections of mice mesenteric arteries loaded for 2 h with 4,5-diaminoflfluorescein (DAF-FM) reveal nitric oxide production after treatment with acetylcholine (Ach 10^−6^ M) or HB (50 µg/mL) and after 30 min of pretreatment with L-NAME (300 µmol/L), counterstained with haematoxylin and eosin (HE). Scale bar, 50 µm. (**F**) (left): representative immunoblots of mice mesenteric arteries treated with HB (100 µg/mL) alone, or HB plus Akt inhibitor or LY294002; (right): semiquantitative analyses, columns are the mean ± SEM of three independent experiments. * *p* < 0.05; ** *p* < 0.01, *** *p* < 0.001.

To investigate the possible vascular molecular mechanisms recruited by HB, we performed a new set of experiments on mice mesenteric arteries, considered the prototype of resistance vessels involved in blood pressure regulation. As shown in Figure 2A, the administration of increasing doses of HB to phenylephrine-pre-constricted mesenteric arteries pre-treated with L-NAME, a well-validated endothelial nitric oxide synthase (eNOS) inhibitor, showed a complete abolition of its vasorelaxant effect, thus suggesting that eNOS enzyme represents the endothelial target molecule necessary to translate the vascular effect of HB. To dissect the molecular mechanism involved, we interrogated one of the most important modulators of eNOS metabolism, Akt, a serine/threonine-specific protein kinase that plays a key role in endothelium-dependent relaxation through the eNOS enzyme. Interestingly, the use of the Akt inhibitor was able to completely block the vasorelaxant effect of HB (Figure 2B). Generally, to activate Akt, some upstream molecules are needed in order to then elicit the activation of nitric oxide production. In this regard, phosphatidylinositol 3-kinase (PI3K) represents one of the most characterized molecules involved in the intracellular activation of eNOS. In our experimental setting, the pre-treatment with LY294002 (a potent chemical inhibitor of PI3K) was able to abolish the vascular action of HB (Figure 2C), whereas AMPK inhibitor did not (Figure 2D); thus, leading us to hypothesize that PI3K/AKT/eNOS-dependent signaling pathway represents one of the possible pathways involved in HB vascular effect. The DAF-FM fluorescence measurement induced by HB was comparable to that obtained with a classical agonist that evokes NO release, such as acetylcholine, and L-NAME pretreatment clearly abolishes endothelial nitric oxide release (Figure 2E). The western blot analyses showed that HB is able, through PI3K and Akt, to positively modulate the Serine 1177 phosphorylation site of eNOS, the most critical activation site of the enzyme that promotes NO production (Figure 2F).

### 3.3. Vascular Evaluation of Most Abundant Single Anthocyanins Included in Healthberry 865^®^ Composition

**Figure 3:**
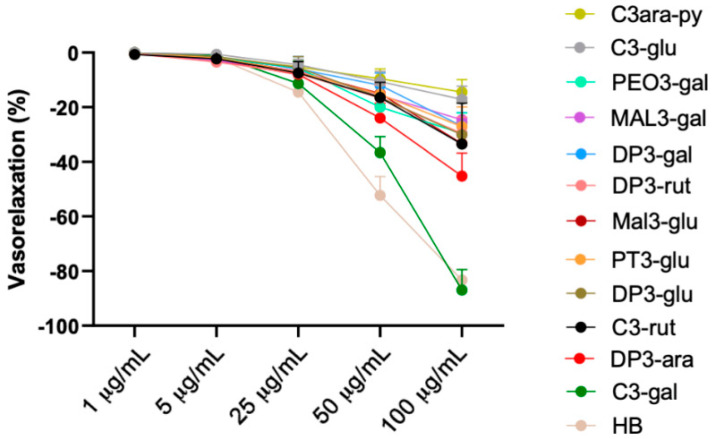
Characterization of vascular action of single anthocyanins. Vascular response of phenylephrine-precontracted mice mesenteric arteries to increasing doses of single anthocyanins, cyanidin-3-arabinopyranoside (C3-ara-py) (*N* = 5), cyanidin-3-glucoside (C3-glu) (*N* = 6), peonidin-3-galactoside (PEO3-gal) (*N* = 3), malvidin-3-galactoside (MAL3-gal) (*N* = 3), delphinidin-3-galactoside (DP3-gal) (*N* = 3), delfinidin-3-rutinoside (DP3-rut) (*N* = 6), malvidin-3-glucoside (MAL3-glu) (*N* = 3), petunidin-3-glucoside (PT3-glu) (*N* = 6), delphinidin-3-glucoside (DP3-glu) (*N* = 8), cyanidin-3-rutinoside (C3-rut) (*N* = 7), delphinidin-3-arabinoside (DP3-ara) (*N* = 5), cyanidin-3-galactoside (C3-gal) (*N* = 3), and HB (*N* = 5) (1–100 µg/mL).

Subsequently, we tested the vascular properties of the of the most abundant and readily available commercially single anthocyanins contained in HB, delphinidin-3-rutinoside (D3-rut), cyanidin-3-rutinoside (C3-rut), delphinidin-3-glucoside (DP3-glu), cyanidin-3-glucoside (C3-glu), petunidin-3-glucoside (PT3-glu), delphinidin-3-galactoside (DP3-gal), peonidin-3-galactoside (PEO3-gal), delphinidin-3-arabinoside (DP3-ara), malvidin-3-galactoside (MAL3-gal), malvidin-3-glucoside (MAL3-glu), cyanidin-3-galactoside (C3-gal), cyanidin-3-arabinopyranoside (C3-arapy) on mice mesenteric arteries (Figure 3).

Interestingly, the evaluation of the possible direct vascular action of C3-rut, C3-glu, DP3-glu, PT3-glu, DP3-glu PEO3-gal, DP3-gal, MAL3-gal, DP3-ara, and MAL-3glu revealed that none of the single anthocyanins were able to evoke a dose-dependent vasorelaxation comparable to that observed after HB administration. A more in-depth analysis of dose–response curves showed that DP3-ara and C3-rut reached the better vasorelaxation compared to the others (about 35–41%) (Figure 3). In contrast, only C3-gal was able to recapitulate the direct vasorelaxant effect of Healthberry 865^®^, evoking a vasorelaxant curve was very similar to that induced by full compound (Figure 3—green).

### 3.4. Cyanidin-3-Galactoside Exerts Vasorelaxant Action Using eNOS Pathway

**Figure 4:**
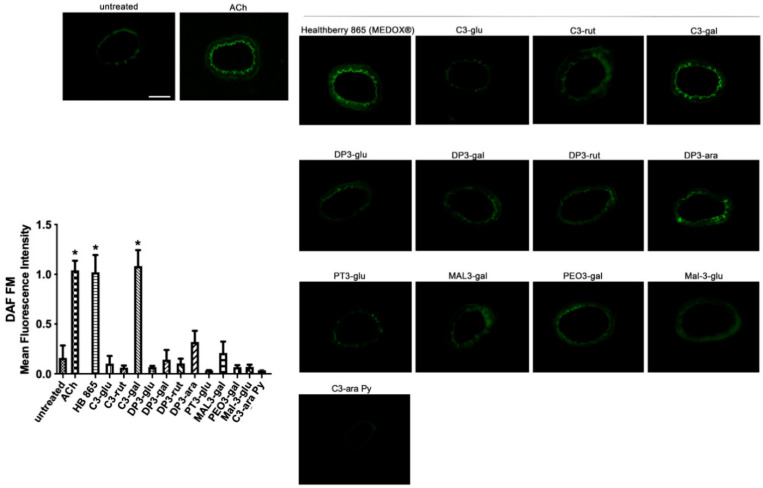
Representative high-power micrographs of 10 µm sections of mice mesenteric arteries loaded for 2 h with 4,5-diaminoflfluorescein (DAF-FM) reveal nitric oxide production after treatment with acetylcholine (Ach 10^−6^ M) or single anthocyanins (50 µg/mL). Scale bar: 100 μm. Bar graph shows the mean fluorescence intensity of *N* = 4 section for each anthocyanin. * *p* < 0.05 versus all.

**Figure 5:**
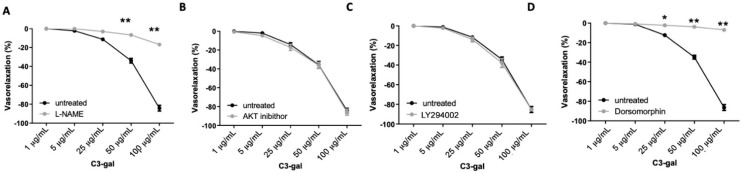
(**A**–**D**) Vascular response of phenylephrine-precontracted mice mesenteric arteries to increasing doses of cyanidin-3-galactoside (**A**) in presence of L-NAME (*N* = 5), (**B**) in presence of Akt inhibitor (*N* = 5), (**C**) in presence of PI3K inhibitor LY294002 (*N* = 5), (**D**) or in presence of dorsomorphin (*N* = 5), a selective AMPK inhibitor. * *p* < 0.05; ** *p* < 0.01.

In order to evaluate the direct vascular action of the single anthocyanins on the modulation of nitric oxide synthase, which is the enzyme involved in HB evoked-vasorelaxation, we performed the analysis and measurement of vessels-derived-nitric oxide after treatment of mesenteric arteries with each anthocyanin. Interestingly, although we observed slight vasorelaxant effects evoked by DP3-gal, C3-rut and DP3-ara, a low signal was obtained in the measurement of NO. In contrast, C3-gal was able to evoke a significant increase of nitric oxide production from vessels, similarly to that observed after HB treatment (Figure 4). Thus, we investigated the possible molecular mechanisms recruited. Of note, the inhibition of eNOS, by L-NAME, significantly reduced the vasorelaxant properties of C3-gal (Figure 5A), thus confirming NO-dependent vasorelaxation. However, differently from what we have observed in HB vascular action, the presence of Akt or PI3K inhibitor did not inhibit C3-gal vascular activity (Figure 5B,C), assuming a different upstream pathway. Thus, we used dorsomorphin, an important selective inhibitor of AMPK. In this condition, C3-gal completely lost its vasorelaxant capability (Figure 5D).

### 3.5. The Antioxidant Vascular Action of Healthberry 865^®^ Is Due to the Combination of the Contained Anthocyanins

**Figure 6:**
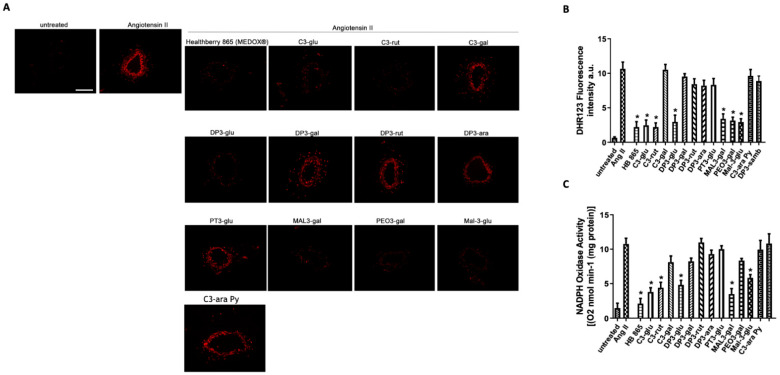
(**A**) Representative high-power micrographs of 10 µm sections of mice mesenteric arteries loaded with a dihydroethidium probe at the concentration of 5 μM. Vessels were pre-treated with single anthocyanins (50 µg/mL) for 1 h and then stimulated with angiotensin II for 15 min prior to the acquisition. Scale bar: 100 μm. (**B**) Measurement of ROS production by DHR123 in vessels treated with single anthocyanins (*N* = 4). (**C**) NADPH oxidase activity in mesenteric arteries exposed to HB or single anthocyanins (*N* = 4). Data are expressed as increase of chemiluminescence per minute. * *p* < 0.05.

Previously, few studies have reported on the antioxidant activities of HB in human subjects [26]. Here, to investigate the capability of HB and their relative single anthocyanins on the modulation of oxidative stress, we performed several methodological approaches, measuring total anti-reactive oxygen species (ROS) capacity and their specific actions on the modulation of the leading machinery of ROS production, the activity of NADPH oxidase enzyme. Our studies, performed on mice mesenteric arteries, revealed that HB has an important anti-oxidative action, as shown by the significant reduction of Angiotensin II-induced ROS formation (Figure 6A). A more in-depth analysis, using single anthocyanins revealed that C3-glu, C3-rut, DP3-glu, MAL3-gal, PEO3-gal, Mal-3-glu are able to reproduce the antioxidant action of Healthberry 865^®^. Accordingly, the biochemical measurement of ROS generation by DHR1,2,3 probe confirm the results obtained with DHE (Figure 6B).

At this point, encouraged by the identification of antioxidant properties of HB and selective anthocyanins, we performed the analysis of NADPH oxidase (NOX) activity after stimulation with angiotensin II, a gold-standard inducer of NOX activation. Our results showed that C3-glu, C3-rut, DP3-glu, MAL3-gal, PEO3-gal, MAL3-glu were able to reduce NOX activity. However, these single anthocyanins resulted in a smaller reduction than evoked by HB (Figure 6C). In fact, C3-glu and MAL3-glu were the most powerful anthocyanins, closer to the potent effect of Healthberry 865^®^.

### 3.6. A Mix of Specific Healthberry 865^®^-Anthocyanins Exert a Powerful Vasorelaxant and Antioxidative Action

**Figure 7:**
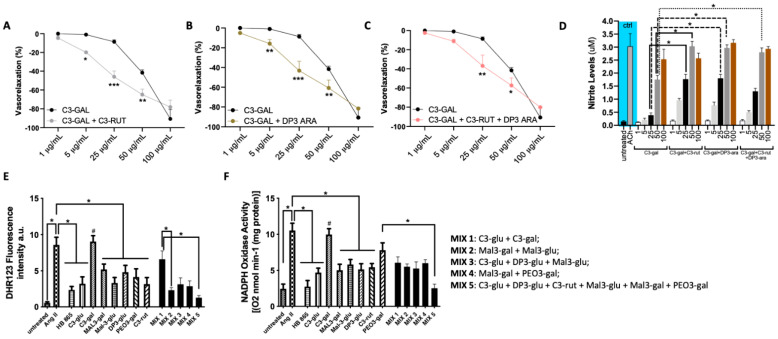
(**A**–**C**) Vascular response of phenylephrine-precontracted mice mesenteric arteries to increasing doses of a combination of anthocyanins mixed with a ratio 1:1. (**A**) cyanidin-3-galactoside (C3-gal) plus cyanidin-3-rutinoside (C3-rut); (**B**) cyanidin-3-galactoside (C3-gal) plus delphinidin-3-arabinoside (DP3-ara); (**C**) cyanidin-3-galactoside (C3-gal) plus cyanidin-3-rutinoside (C3-rut) plus delphinidin-3-arabinoside (DP3-ara); *N* = 3 for each curve. (**D**) Nitrite measurement in organ bath after stimulation with increasing doses of different mix (*N* = 3 for each treatment). (**E**,**F**) Measurement of ROS production by DHR123 in vessels (*N* = 4 for each treatment) treated with anthocyanins mixture and (**F**) NADPH oxidase activity (*N* = 4 for each treatment). Data are expressed as increase of chemiluminescence per minute. * *p* < 0.05; ** *p* < 0.01, *** *p* < 0.001; ^#^ *p* < 0.05.

Based on the results obtained up until now, we wanted to investigate the possible effects on vasorelaxation and antioxidative action of a mix of different Healthberry 865^®^-anthocyanins. To pursue this goal, we combined C3-galactoside, the most potent vasorelaxant anthocyanin, with C3-rut, DP3-ara, C3-rut, or with DP3-ara/C3-rut in a triple combination, normalizing their relative concentration for each dose–response curve in order to obtain a combination with two anthocyanins a ratio of ½:½, and for three, 1/3:1/3:1/3. Surprisingly, in combination with the C3-rut, we observed an improvement of the vasorelaxant curve, which, although reaching the same maximal point of C3-gal alone, showed a significant increase of middle points (at 5 and 50 ug/mL) (Figure 7A–C). Interestingly, the measurement of nitrite concentration—the stable breakdown of nitric oxide (in the organ bath) showed a significant improvement of combinations C3-gal/C3-rut and C3-gal/DP3-ara at 25 and 50 ug/mL in comparison to C3-gal alone (Figure 7D).

To evaluate the role on oxidative stress, we assessed the action or different mixes which acted on the modulation of oxidative stress, mixing different anthocyanins: MIX 1: C3-glu + C3-gal; MIX 2: Mal3-glu + Mal3-gal; MIX 3: C3-glu + DP3-glu + Mal3-glu; MIX 4: Mal3-gal + PEO3-gal; MIX 5: C3-glu + DP3-glu + C3-rut + Mal3-glu + Mal3-gal + PEO3-gal. Interestingly, the measurements of both total ROS production and NADPH oxidase activity revealed the highest efficacy of MIX 5 (Figure 7E,F).

### 3.7. Healthberry 865^®^ Reduces Oxidative Stress and Improves NO Bioavailability in Human Dysfunctional Vessels

In order to assess also in human, the effects observed in ex-vivo on mice mesenteric arteries, we have evaluated the action of HB on the human superior thyroid artery (STA), obtained from patients undergoing carotid revascularization surgery. In particular we assessed its effect on vessel rings obtained from hypertensive patients under diuretic treatment to avoid possible confounding factors related with pharmacological treatments known to improve endothelial function and NO release (such as, RAAS antagonist). Clinical characteristics are reported in supplementary Table S2.

**Figure 8:**
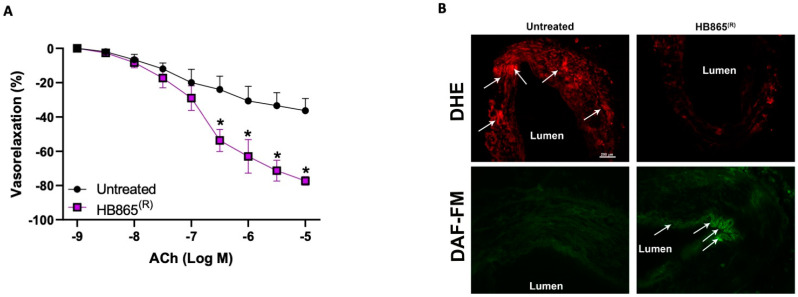
(**A**) Dose–response curves of relaxation of human superior thyroid artery (STA) collected from hypertensive and dyslipidemic patients in response to increasing doses of acetylcholine (ACh) alone, or after preincubation with HB865^®^ for 1 h at the dosage of 50 μM. The response obtained was expressed as the percentage. Data are given as mean ± SEM (*N* = 4); (**B**) dihydroethidium (DHE) and DAF-FM staining of human vessels untreated or treated with HB865^®^ for 1 h. Scale bar: 200 μm. * *p* < 0.05.

At baseline, STA presented an important endothelial dysfunction, as shown by the altered acetylcholine-evoked vasorelaxation (Figure 8A). Of note, the treatment with 50 ug/mL of HB for 1 h was able to significantly improve the altered endothelial vasorelaxation. The measurement of total oxidative stress revealed that after HB treatment there was a significant reduction of ROS in the vessels (Figure 8B). However, it is even more surprising that after HB stimulation there is a considerable increase of NO-production, which perfectly reflects the improvement of vasorelaxant response.

## 4. Discussion

In this study, for the first time, we have characterized the direct vascular action of HB on different vascular districts, demonstrating its direct endothelium-dependent vasorelaxant capacity in a dose-dependent manner. Activation of PI3K/Akt/eNOS is required for its vascular action since the pharmacological inhibition of this intracellular signaling completely abolished its vascular action. Moreover, it exerts an important antioxidative action, reducing ROS production and inhibiting NADPH oxidase activity. Interestingly, C3-gal, an anthocyanin included in HB composition, mediates its direct vascular action, whereas other anthocyanins, such as C3-glu, C3-rut, MAL3-glu, MAL3-gal, and PEO3-gal mediate the antioxidant action of HB on vascular tissue.

Anthocyanins are water-soluble compounds, mainly concentrated in fruits and vegetables, and are consistently reported to exert several beneficial effects on human health [27,28]. Interestingly, it seems that different anthocyanins may possess different bioactivities on cardiovascular system [29,30,31]. Unfortunately, CVD is common both in industrialized and non-industrialized countries, and is responsible for millions of deaths annually, worldwide [32]. The main objective of classical medicine is to apply a pharmacological therapy able to contain the progression of CVDs without adversely affecting quality of life. However, another pivotal goal is to prevent the development of incidence of stroke, myocardial infarction, and heart failure, which often cannot be accomplished with the classical pharmacological therapy and are prevalently addressed by lifestyle changes as in subjects with pre-hypertension (pre-HTN). Indeed, pharmacological therapy can be applied upon a diagnosed CVD; thus, limiting the possibility of intervening at a preventative stage. This gap can be filled by the use of substances able to foster favorable and protective vascular responses and with relatively low risk of side effects. To date, interest in nutraceuticals for cardiovascular prevention was particularly stimulated after the observations of a close association between their consumption and a reduced cardiovascular event rate [33]. An increasing amount of scientific evidence demonstrates their potential in cardiovascular prevention and, recently, anthocyanins have also garnered increasing attention due to their specific cardiovascular properties [34,35]. Although a different beneficial action of anthocyanins has been reported, a direct possible vascular action of HB or single anthocyanins has never been investigated.

Thus, the aim of our study was to assess the vascular properties of the HB compound that contained a defined mixture of purified anthocyanins, to exclude possible interactions of other natural-derived components. To pursue this goal, we performed ex-vivo vascular reactivity studies in mice and human vessels, since this method represents a well-validated scientific approach that allows to characterize the possible role of several compounds on different districts of the vascular system, allowing to investigate the molecular mechanisms involved. Although there is enormous potential of the ex-vivo vascular reactivity study, it is important to underline that, like all ex-vivo approaches, this system bypasses the process of absorption, distribution, and circulation to which a substance is subjected in vivo; nevertheless, it allows expanding the knowledge and defining the possible field of application of new compounds and molecules [36,37,38].

In our experimental condition, a dose-dependent administration of HB evokes specific endothelial vasorelaxation, in both conduit and resistance arteries, such as aorta, carotid, and in femoral and mesenteric arteries, respectively. Based on the effect evoked by HB on resistance arteries, and considering that the alteration of endothelium-dependent vasodilation in resistance arteries, but not in the conduit artery, has been associated with a 5-year risk of a composite end-point of death, myocardial infarction, or stroke, independent of major cardiovascular disease risk factors [39], we focused our studies on mice mesenteric arteries, which better reproduce the function of human resistance arteries. The eNOS enzyme represents the main downstream protein through which the HB-evoked endothelial vasorelaxation. The mechanism recruited by HB for modulation of the vascular tone was represented by the recruitment of PI3K and Akt, two important modulators of eNOS signaling [40]. Accordingly, the assessment of nitric oxide production by DAF-FM clearly revealed the capability of HB to induce nitric oxide production at the endothelial layer, reproducing the action evoked by ACh, the gold standard molecule used to assess endothelial-derived nitric oxide production. These encouraging results have led us to investigate the possible specific anthocyanins able to reproduce the vascular action of Healthberry 865^®^. To pursue this goal, we assessed the vascular function of the main Healthberry 865^®^-concentrated anthocyanins.

In this regard, although several studies have evaluated the possible vasoprotective and vasoactive functions of single anthocyanins, until now, nobody has assessed their capability of evoking a direct vascular action. The analysis of vascular function revealed that the only anthocyanins that possess similar effects to HB are represented by cyanidin-3-galactoside. The inhibition of nitric oxide confirms its NO-dependent action; thus, this led us to hypothesize that this anthocyanin recapitulates the vasoactive action of Healthberry 865^®^. However, a deeper analysis revealed that the inhibition of PI3K or Akt did not evoke any action on its vasorelaxant properties. Surprisingly, the inhibition of AMPK was able to completely abolish the action of cyanidin-3-galactoside. Although it is difficult to understand and explain this possible differential action of cyanidin-3-galactoside from Healthberry 865^®^, we cannot exclude that the administration of the entire compound could lead to the potential generation of additional novel compounds, possibly derived from interactions between different anthocyanins. Of course, this result will lead us to evaluate the possible interactions between anthocyanins in a new biochemical study in the future, in order to better understand their potential mechanisms of action and any metabolites that may be generated. After all, following in vivo administration of an anthocyanin mixture, it was shown that what is detected in the blood stream is very different from the molecular structure typical of anthocyanins, since they are metabolized by the microbiome or other liver enzymes [41].

As suggested in 2005 by Bell and Gochenaur, anthocyanin-rich extracts also possess powerful antioxidant action [42], making them of potential importance to cardiovascular diseases, such as atherosclerosis, hypertension, and diabetes, which are extensively characterized by an increase of ROS production [43,44,45]. In such conditions of excess vascular and extravascular production of ROS, there is an impairment of NO bioavailability, leading to endothelial damage and dysfunction. Clearly, factors that can enhance or protect the endothelial NO system, or scavenge and inactivate ROS, have the potential to exert an important cardiovascular protection. Our studies revealed an important antioxidant action of HB on mice mesenteric arteries stimulated with angiotensin-II, which is able to evoke ROS generation in vasculature [46], since HB completely abolished ROS production, thus clearly demonstrating the important antioxidant action of this mixture of anthocyanins. It is well known that oxidative stress can be reduced by different biochemical processes, through a scavenger action or modulating the main ROS intracellular complex, NADPH oxidase enzyme. Interestingly, HB treatment is able to significantly reduce the amount of oxidative stress. In a similarly manner, six specific single anthocyanins, C3-glu, C3-rut, DP3-glu, MAL3-glu, MAL3-gal, and PEO3-gal included in the mixture, revealed important antioxidant properties. Even more interesting is that the activity of NADPH oxidase, the main endogenous pro-oxidant machinery, was completely blunted by Healthberry 865^®^, and, to a lesser extent, by C3-glu, C3-rut, DP3-glu, MAL3-gal, and MAL-3glu, demonstrating that these single anthocyanins confer to HB a higher vascular antioxidant action.

Following our future perspective, aimed at selecting the best composition of anthocyanins capable of exerting vascular protection, we have assessed the possible actions both on endothelial vasorelaxation and on antioxidant action of a different combination of single Healthberry 865^®^ anthocyanins. The combination of C3-gal and DP3-ara was able to evoke a significant improvement of endothelial vasorelaxation, ameliorating the dose–response of mesenteric arteries in comparison to C3-gal alone, which as reported exerted the better vasorelaxant action, relative to all other anthocyanins. Interestingly, the action of the combination of C3-gal and DP3-ara is due to a boost of nitric oxide release at lower doses compared to C3-gal alone, suggesting a possible interaction between these anthocyanins or a generation of novel metabolites able to evoke a more powerful vascular action.

Finally, to put the foundation on the potent beneficial effects of anthocyanins in human experimental models, using dysfunctional human vessels, we demonstrated that HB is able to not only significantly improve endothelial-dependent vasorelaxation, but we also confirmed that its action is mediated by the powerful antioxidant action, which, in turn, increases nitric oxide bioavailability. The ability to maintain nitric oxide bioavailability represents the main determinant of the incidence and progression of several cardiovascular diseases.

## 5. Conclusions

Our data demonstrate the direct role of Healthberry 865^®^ on the modulation of vasculature, on both the vasorelaxation and on oxidative stress. In particular, its capability to induce nitric oxide production and to reduce the activity of one of the most important machinery or ROS generation, NADPH oxidase, strongly supports the potential beneficial effects on the cardiovascular system.

## 6. Limitation of the Study

Thus far, although promising data from ex vivo mice and human arteries fully support the concept that a purified and defined blend of anthocyanins from blueberries and black currants can help contain or prevent the onset of CVD-associated vascular dysfunction, further studies will be needed to investigate the in vivo effects of Healthberry 865^®^. The complete characterization of the absorption, distribution, metabolism, and excretion of HB will be necessary to define its performance and the pharmacological activity of the compound. Moreover, numerous ad-hoc studies designed to identify in vivo dosages and effects evoked by HB and its related-single anthocyanins will be necessary in order to delineate the real beneficial potential of this new purified anthocyanin blend.

## Data Availability

The data presented in this study are available upon request from the corresponding author. The data are not publicly available due to the development of the patent application.

## Supplementary Materials

The following are available online at https://www.mdpi.com/article/10.3390/antiox10081191/s1, Figure S1: HPLC analysis of Healthberry 865, Figure S2: EICS (extracted ion chromatograms), covering all relevant anthocyanins, part 1, Figure S3: EICS (extracted ion chromatograms), covering all relevant anthocyanins, part 2, Table S1: Results for the quantitation of all relevant anthocyanins., Table S2: Clinical characteristics of patients.
