# Supplementary material for: Healthberry 865® and Its Related, Specific, Single Anthocyanins Exert a Direct Vascular Action, Modulating Both Endothelial Function and Oxidative Stress
Source: Antioxidants (Basel). 2021 Jul 26;10(8):1191. doi: 10.3390/antiox10081191 (PMC8388872; doi:10.3390/antiox10081191)
Supplement: Supplementary file 1 [file antioxidants-10-01191-s001.zip › antioxidants-1300731-supplementary.pdf]

antioxidants

MDPI

Supplementary Materials: Healthberry 865® and Its Related, Specific, Single Anthocyanins Exert a Direct Vascular Action, Modulating Both Endothelial Function and Oxidative Stress

Albino Carrizzo [1,2], Rosario Lizio [3], Paola Di Pietro [1], Michele Ciccarelli [1], Antonio Damato [2], Eleonora Venturini [2], Patrizia Iannece [1], Eduardo Sommella [4], Pietro Campiglia [4], Philipp Ockermann [5] and Carmine Vecchione [1,2,*]

[1] Cardiovascular Research Unit—Department of Medicine and Surgery, University of Salerno, 84081 Baronissi, Italy; albino.carrizzo@gmail.com (A.C.); pdipietro@unisa.it (P.D.P.); mciccarelli@unisa.it (M.C.); piannece@unisa.it (P.I.)
[2] Laboratory of Vascular Physiopathology—I.R.C.C.S. Neuromed, 86077 Pozzilli, Italy; antonio.damato85@gmail.com (A.D.); eleonora.venturini94@libero.it (E.V.)
[3] Evonik Operations GmbH, Rodenbacher Chaussee 4, 63457 Hanau, Germany; rosario.lizio@evonik.com
[4] Department of Pharmacy, University of Salerno, 84084 Fisciano, Italy; esommella@unisa.it (E.S.); pcampiglia@unisa.it (P.C.)
[5] Institute for Tissue Engineering and Regenerative Medicine, Universität Würzburg, Josef-Schneider Straße 2, 97080, Würzburg, Germany; philipp.ockermann@uni-wuerzburg.de
* Correspondence: cvecchione@unisa.it

Citation: Carrizzo, A.; Lizio, R.; Pietro, P.D.; Ciccarelli, M.; Damato, A.; Venturini, E.; Iannece, P.; Sommella, E.; Campiglia, P.; Ockermann, P.; Vecchione, C. Healthberry 865® and Its Related-Specific Single Anthocyanins Exert a Direct Vascular Action Modulating Both Endothelial Function and Oxidative Stress. *Antioxidants* **2021**, *10*, 1191. https://doi.org/10.3390/antiox10081191

Academic Editors: Rosario Ammendola and Fabio Cattaneo

Received: 30 June 2021
Accepted: 20 July 2021
Published: 26 July 2021

Publisher's Note: MDPI stays neutral with regard to jurisdictional claims in published maps and institutional affiliations.

Copyright: © 2021 by the authors. Submitted for possible open access publication under the terms and conditions of the Creative Commons Attribution (CC BY) license (http://creativecommons.org/licenses/by/4.0/).

Anthocyanins quantification by HPLC-MS method

By using an HPLC-MS method it was possible to detect and quantify the present anthocyanins in Healthberry 865. This was done due to external calibration by using single anthocyanins as reference material and therefore being more specific and precise. As their purity was unknown, every single anthocyanin was analyzed by HPLC-MS prior to the analysis.

All the reference materials contained trace amounts resulting in little peak responses. DP3-rut contained DP3-glu and could therefore not be used for direct quantification. In order to get a result, the amounts of DP3-glu and DP3-rut were estimated by their areas: area of the peaks formed by DP3-glu and DP3-rut = 100 %. DP3-ara could not be detected in the reference material but in Healthberry 865.

Table 1 presents the results for the quantitation of all relevant anthocyanins. Figure 1 presents the findings as graphical summary.

Anthocyanin	Healthberry HPLC MS [% w/w]
DP3-gal	1.65
C3-gal	1.21
C3-glu	1.57
C3-arapy	2.37
C3-rut	3.19
PT3-glu	2.03
PEO3-gal	0.20
PEO3-glu	0.96
MAL3-gal	0.16
MAL3-glu	5.13
DP3-glu	-
DP3-ara	-
DP3-rut	3.18
PT3-gal	0.48

**PEO3-ara**	0.61
**Sum HPLC**	22.74
**Sum UV**	39.07

Table S1. Results for the quantitation of all relevant anthocyanins.

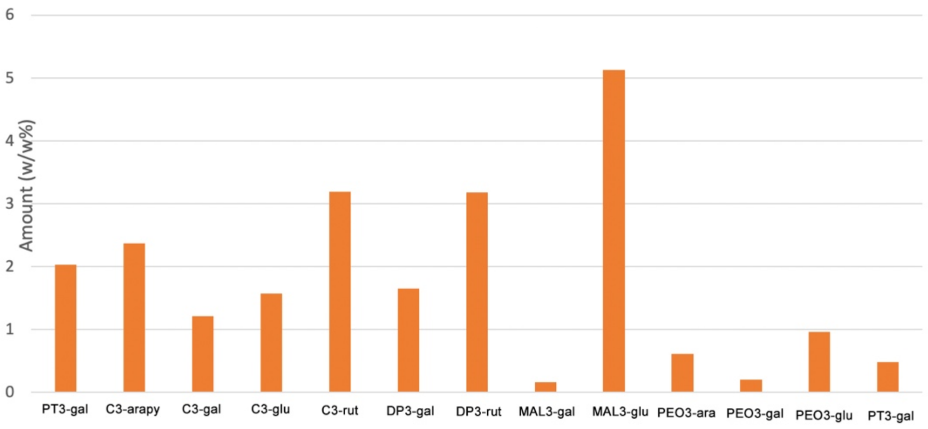

Figure S1. HPLC analysis of Healthberry 865.

Method and Results.

**Analytical Method**	**HPLC-UV/MS**
**Instrument**	Accela 1250 equipped with an Orbitrap Elite (Thermo Fischer)
**Detection**	ESI positive, UV (520 nm)
**Mass Range**	400 – 650 u
**Analytical Column**	250x4 mm EC NUCLEODUR π2, 5 µm (Macherey-Nagel, REF
**760625.40)**	
**Eluent A**	H2O + Formic Acid (94/6, v/v)
**Eluent B**	Methanol
**Gradient**	5%B – in 30 min - 95% - 10 min isocratic
**Posttime**	10 min
**Flow**	0.75 mL/min
**Temperature**	65°C
**Injection volume**	1 µL
**Diluent**	H2O + Formic Acid (94/6, v/v)
**Sample Preparation**	approx. 1 mg/mL in Diluent

The method was not developed by the laboratory itself. A commercial analytic column for detection of anthocyanins was used, provided by the manufacture: MACHEREY-NAGEL GmbH & Co. KG, Neumann-Neander-Str. 6–8, 52355 Düren/D.

Literatur: "Bestimmung von Anthocyanen in Fruchtsäften per HPLC – UV", H. R. Wollseifen, Düren/D, T. Kretschmer; http://ftp.mnnet.com/deutsch/Poster/Chromatographie/Poster_Anthocyane_Fruchtsaefte_www.pdf (12/18/2019,14:38 (MEZ))

However, the amount of formic acid in the eluent system was increased to increase separation of isomeric compounds. The resulting chromatograms of the analysis of the sample materials Healtberry 865 are shown in figure 2 to 3. As the purity and/or the

content of most of the reference materials was unknown, each reference material was analyzed by HPLC-MS.

All the reference materials contain trace amounts resulting in little peak responses. As the content of the reference materials is unknown, there is already a certain error in the quantitation results. An analytical method itself also provides a certain error. Therefore, the little peak responses could be neglected in this case.

DP3-rut contained DP3-glu. As the calibration solutions contains all the reference materials the amount of DP3-glu and the amount of DP3-rut in the calibration solutions are unknown. Therefore, the calibration of these two anthocyanoses was not possible based on the current data.

A different sample of DP3-rut also contained a certain, unknown amount of DP3-glu. Therefore, the calibration of DP3-rut was not possible based on the current data. In order to get a result, the amounts of DP3-glu and DP3-rut were estimated by their areas: area of the peaks formed by DP3-glu and DP3-rut = 100 %. This basic calculation only provides a rough estimation, as the response factors of these two molecules are unknown.

DP3-ara could not be detected in the reference material but in the sample materials Healthberry 865.

The quantitation of the anthocyanins in Healthberry 865 by HPLC-MS was accomplished by an external calibration with the given reference materials. As the purity and/or the content of most of these reference materials is unknown, the quantitation results for these compounds are based on an assumed content of 100 %.

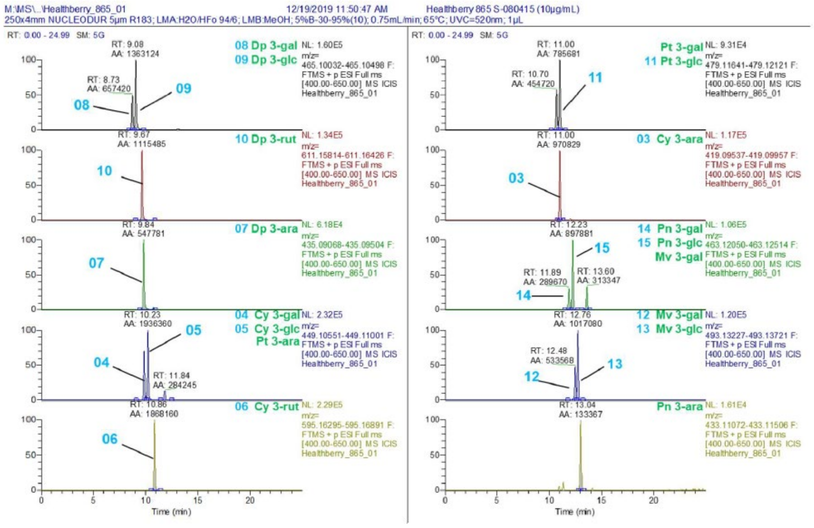

Figure S2. EICS (extracted ion chromatograms), covering all relevant anthocyanins, part 1.

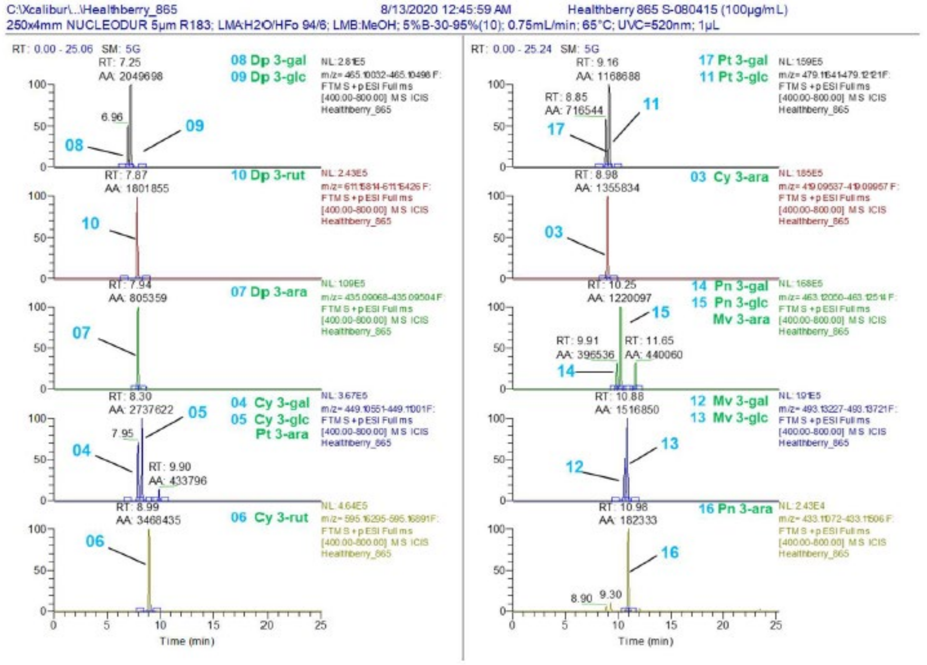

Figure S3. EICS (extracted ion chromatograms), covering all relevant anthocyanins, part 2.

List of supplied anthocyanins.

No.	Product Description	Provider
1	Healthberry 865	Evonik Industries
3	C3-gal	Polyphenols
4	C3-glu	Polyphenols
5	C3-rut	Polyphenols
6	DP3-arapy	AOBIOUS
7	DP3-gal	Sigma Aldrich
8	DP3-glu	Polyphenols
9	DP3-rut	Polyphenols
10	PT3-glu	Polyphenols
11	MAL3-gal	Sigma Aldrich
12	MAL3-glu	Polyphenols
13	PEO3-gal	Polyphenols

Clinical characteristics	Subjects (N=4)
**Age average, y**	56,1 ± 4
**Risk factors, n (%)**	
**Hypertension**	100%
**Dyslipidemia**	0
**Cardiopathy**	0
**Diabetes mellitus**	0
**Smoker**	25%
**COPD**	0
**Hepatopathy**	0
**Dysthyroidism**	0

**Previous surgery**	25%
**Medications, n (%)**	
**Diuretics**	100%
**ASA**	0
**ACE inhibitors**	0
**Statins**	0

Table S2. Clinical characteristics of patients.
